# Authors’ response: Statistical methodology critique and alternative approaches in H5Nx avian influenza seroprevalence study among French cats

**DOI:** 10.2807/1560-7917.ES.2025.30.15.2500247

**Published:** 2025-04-17

**Authors:** Brandon Hayes, Pierre Bessière, Jean-Luc Guérin

**Affiliations:** 1 IHAP, Université de Toulouse, INRAE, ENVT, Toulouse, France

**Keywords:** Epidemiological methodology, logistic regression, seroprevalence analysis, statistical assumptions

**To the editor:** We appreciate our colleague’s interest in our study and their engagement with our employed methodology. However, we respectfully disagree with the assertion that our approach violates multiple fundamental assumptions of Firth regression. Firth’s penalised maximum likelihood estimation was designed to address bias from small samples as well as handle complete or quasi-complete separation in data [1-3]. Several of the cited assumptions—including ‘no perfect separation’ and ‘sufficient sample size’—are not only not violated but rather are directly addressed through this approach.

In regression modelling, the most important assumption is validity of the model itself: that is, whether the chosen predictors can explain variation in the outcome [4]. This is followed by additivity, linearity and, for the residuals, independence, equal variance and normality. Our model satisfies this principal condition through focusing on biologically plausible predictors of H5 serostatus in cats: environmental exposure through proxies of ownership and hunting behaviour, and regional highly pathogenic avian influenza (HPAI) outbreak risk in poultry.

The imbalanced sampling distribution, reflecting a targeted sampling approach that prioritised departments classified as at-risk, can indeed be a concern from an external validity perspective. However, this imbalance is not a violation of regression assumptions [5], and is even explicitly addressed in our model’s structure by including the HPAI risk status of the departments as a covariate.

We agree that independence of observations is a core tenant of logistic regression, and that potential clustering could exist within or between departments and serve as a source of dependence. Although our data were too sparse to assess 33 department-level effects, in future analyses, mixed-effect or spatial regression models could be employed to account for this more explicitly.

Following the suggested analytical techniques, we examined monotonic relationships. Stray status showed a weakly significant association with H5 seropositivity (Spearman’s ρ = 0.074; p = 0.047), while hunting behaviour among owned cats did not. When combined into a composite variable of both stray status and hunting frequency (believed to represent degrees of environmental exposure), a slightly stronger and still significant correlation was seen (ρ = 0.090; p = 0.016). Mutual information analysis supported these conclusions, with hunting behaviour and the composite ‘stray + hunt’ variable (for overall environmental exposure) suggested to be the most informative predictors of serostatus (Table).

**Table:** Mutual information ranking scores

Variable	MI score
Hunting behaviour	0.0165
Stray + hunt interaction	0.0026
Stray status	0.0021
Department at risk	0.0020
Department	0.0014

MI: mutual information.

However, although these techniques are well suited for exploring this dataset, they are not a substitute for regression modelling when the goal is to estimate the strength and direction of associations. Despite the potential for some degree of violation of the assumption of independence due to spatial clustering (with associated potential for inflated type I errors and/or exaggerated effects size), when used with appropriate caution and transparency, regression can still provide insights into the factors associated with seropositivity in our study [6].

Conducting robust and accurate data analyses is paramount to advancing the quality of research in our field. This peer critique has provided a valuable opportunity to reflect on our methodology choices and deepen our understanding of the complexities inherent to serosurvey data. We thank our colleagues for the opportunity to engage in this dialogue and help elevate the scientific standard, and we look forward to further discussion as it arises.
